# Visual mismatch negativity: a predictive coding view

**DOI:** 10.3389/fnhum.2014.00666

**Published:** 2014-09-16

**Authors:** Gábor Stefanics, Jan Kremláček, István Czigler

**Affiliations:** ^1^Translational Neuromodeling Unit, Institute for Biomedical Engineering, University of ZurichETH Zurich, Zurich, Switzerland; ^2^Laboratory for Social and Neural Systems Research, Department of Economics, University of ZurichZurich, Switzerland; ^3^Department of Pathological Physiology, Faculty of Medicine in Hradec Králové, Charles University in PragueHradec Králové, Czech Republic; ^4^Research Center for Natural Sciences, Institute of Cognitive Neuroscience and Psychology, Hungarian Academy of SciencesBudapest, Hungary

**Keywords:** EEG, ERP, perceptual learning, predictive coding, prediction error, repetition suppression, stimulus specific adaptation, visual mismatch negativity

## Abstract

An increasing number of studies investigate the visual mismatch negativity (vMMN) or use the vMMN as a tool to probe various aspects of human cognition. This paper reviews the theoretical underpinnings of vMMN in the light of methodological considerations and provides recommendations for measuring and interpreting the vMMN. The following key issues are discussed from the experimentalist's point of view in a predictive coding framework: (1) experimental protocols and procedures to control “refractoriness” effects; (2) methods to control attention; (3) vMMN and veridical perception.

## Introduction—what is visual MMN and what is it good for?

Current theories of visual change detection emphasize the importance of focal attention to detect changes in the visual environment (Rensink, 2002; Simons and Rensink, 2005). However, an increasing body of studies shows that the human brain is capable of detecting even small visual changes, especially if such changes violate automatic (non-conscious) expectations (based on repeating experiences). In other words, our brain automatically represents statistical regularities of the environment and registers “surprising” events. Since the discovery of the mismatch negativity ERP component, the majority of research in the field has focused on auditory deviance detection, operating outside the focus of active attention. Historically, change detection indexed by the MMN was thought to be primarily an auditory phenomenon (Näätänen et al., 2001), hearing being a “temporal” sensory modality. However, substantial evidence has accumulated suggesting that automatic mechanisms of change detection operate in the visual modality too.

The system generating the auditory MMN has been referred to as a “primitive system of intelligence” by the discoverer of the MMN response (Näätänen et al., 2001). This system organizes the auditory input by extracting the common invariant patterns shared by a number of acoustically varying sounds, anticipates the events of the immediate future in the absence of attention, and even manifests simple concept formation. In a general framework of human cognition Kahneman (2011) postulated two general systems underlying information processing. System 1 is automatic and fast, and works without effort of voluntary control, whereas System 2 uses attention to carry out effortful mental activities^1^. He describes System 1 as “effortlessly originating impressions and feelings that are the main sources of the explicit beliefs and deliberate choices of System 2” and identifies automatic change detection (“Orient to the source of a sudden sound”) as an automatic activity of System 1 which is capable of generating complex patterns of ideas by extracting regularities from the environment. In Kahnemann's framework, the main function of System 1 is to maintain and update our model of the world, which represents what is normal in it, i.e., what is predictable based on past events. The visual MMN can be described as the electrophysiological correlate of the automatic detection of *unpredicted* changes in our visual environment carried out by System 1.

In MMN paradigms short term predictive representations of environmental regularities are thought to be formed based on the observed likelihood of frequently repeating events (standard). Implicitly learned statistical regularities serve as a basis to automatically detect rare events (deviant) which do not match predictions. Recent modeling studies (Lieder et al., 2013a,b) suggest that the (auditory) MMN reflects approximate Bayesian learning of sensory regularities, and that the MMN-generating process adjusts a probabilistic model of the environment according to mismatch responses (MMRs) (prediction errors). The *MMN response is widely considered as a perceptual prediction error signal* (Friston, 2005; Garrido et al., 2008, 2009; den Ouden et al., 2012; Stefanics and Czigler, 2012)—a member of a family of prediction errors, which include perceptual, higher cognitive, and motivational prediction errors.

The notion that automatic change detection in the visual modality does not operate only at the level of simple sensory features such as color (Czigler et al., 2002, 2004, 2006a; Horimoto et al., 2002; Mazza et al., 2005; Kimura et al., 2006b; Liu and Shi, 2008; Grimm et al., 2009; Thierry et al., 2009; Czigler and Sulykos, 2010; Müller et al., 2010; Mo et al., 2011; Stefanics et al., 2011), line orientation (Astikainen et al., 2004, 2008; Czigler and Pató, 2009; Flynn et al., 2009; Kimura et al., 2009, 2010a, 2006b; Czigler and Sulykos, 2010; Sulykos and Czigler, 2011), or spatial frequency (Heslenfeld, 2003; Kenemans et al., 2003, 2010; Maekawa et al., 2005, 2009; Sulykos and Czigler, 2011), but also at higher cognitive levels, has been supported by several visual MMN studies. Recent studies demonstrated that object-based irregularities are automatically detected by the visual system (Müller et al., 2013), as well as irregular lexical information (Shtyrov et al., 2013). Another recent study showed that visual mismatch negativity (vMMN) can be elicited both by real and illusory brightness changes (Sulykos and Czigler, 2014). vMMN was also evoked by changes in abstract attributes (if..then conditional probability) of simple geometric patterns (Stefanics et al., 2011), but also by changes in attributes of complex natural stimuli such as laterality of body parts (Stefanics and Czigler, 2012), or socially more relevant stimuli such as facial emotions (Susac et al., 2004, 2010; Zhao and Li, 2006; Astikainen and Hietanen, 2009; Chang et al., 2010; Kimura et al., 2012; Stefanics et al., 2012; Fujimura and Okanoya, 2013), and facial gender (Kecskés-Kovács et al., 2013b). These observations are well in line with theories of generative models (for reviews, see Kimura et al., 2011; Winkler and Czigler, 2012; Clark, 2013) which posit that unpredicted stimulus attributes evoke mismatch signals (prediction errors) which in turn modifies predictions pertaining to the given attributes.

Although studying both visual and auditory mismatch processes rests on the common principle that extraction of statistical regularities in characteristics of many environmental events can be probed indirectly by recording the MMN response to events which violate such regularities, there are also important methodological differences between visual and auditory mismatch paradigms. For example, to minimize attentional components in ERPs evoked by events in auditory MMN experiments, often a separate visual task is used to engage the attention of participants, thus MMN-evoking stimuli are task-independent and assumed to be unattended. Due to the relative dominance of vision over hearing, primary visual tasks are useful in auditory studies. However, visual MMN studies should also use visual tasks instead of auditory tasks to effectively minimize attentional effects in processing of MMN-evoking stimuli. Here we provide a brief summary of some of the important methodological approaches and their rationale which we believe should be taken into account when one designs a visual MMN protocol and interprets its results.

MMN is often elicited by rare events embedded in a series of frequently repeating events. It is important to emphasize that labeling an event as “surprising,” “unexpected,” or “improbable” can be based on probabilities learned over shorter or longer time scales. Regularities (i.e., probability structure of events) established in MMN/vMMN paradigms exist over relatively short time scales, in the range of 4–15 s in the auditory modality (Mäntysalo and Näätänen, 1987; Cowan et al., 1993; Ulanovsky et al., 2004), and probably less in vision (Astikainen et al., 2008) and have been suggested to be supported by short-term synaptic plasticity (Garrido et al., 2009; Kujala and Näätänen, 2010). The possibility of multiple short-term mechanisms has led to a rather long but not particularly productive debate on the processes underlying the MMN, usually labeled as the “refractoriness” issue. The contribution of the repetition effect to the differential activity evoked by the rare stimulus, i.e., the “refractoriness” issue, will be discussed in Section Memory mismatch and refractoriness.

MMN is usually observed when a “surprising,” “unexpected,” “unpredicted,” or “infrequent” event occurs. It is important to point out that in the context of (v)MMN research, none of these terms refers to processes requiring attention. Registration of the change in likelihood of task-irrelevant environmental events happens in the absence of attention or without conscious effort (Näätänen et al., 2001, 2007, 2010). One prerequisite for such a “surprise” is that the neural populations which generate the MMN have extracted a statistical regularity from the sequence of environmental events, so that it has become able to detect events which deviate from the regular. Surprise can thus only occur if some kind of a prediction has been formed a priori. Although most MMN experiments employ sequential regularities, recent evidence indicates that the human perceptual system implicitly encode non-sequential stochastic regularities too and keep track of the *uncertainty* induced by apparently random distributions of sensory events (Garrido et al., 2013). vMMN paradigms usually employ attention-demanding primary tasks to ensure that activity of conscious attentional mechanisms is not superimposed on mismatch activity. A variety of primary tasks have been used in different studies, which will be discussed in Section Visual MMN and attention.

At the outset of vMMN research, studies focused on individual features (color, spatial frequency, orientation, movement direction, etc.); later vMMN has been investigated for feature conjunctions, object-related deviances and the violation of sequential regulations. Furthermore, an increasing number of studies show, that vMMN is also sensitive to higher-order deviances and correlates with behavioral measures. Importantly, the features defining the contents of automatic expectations can be not only simple physical, but more abstract properties too, even socially relevant signals such as facial emotions. Thus, mechanisms underlying the vMMN are able to support flexible categorization processes (Czigler, 2013). The relationship between visual mismatch and behavior is discussed in Section The link between vMMN, veridical perception, and behavior.

According to the hierarchical predictive coding framework veridical perception is supported by neural processes optimizing probabilistic representations of the causes of sensory inputs (Friston, 2010). The continuous interaction between top-down flow of predictions and bottom-up flow of prediction errors keeps our internal model of reality up-to-date. Here we argue that the visual MMN response is a “special case” of the ubiquitous prediction error signals that support our internal model of reality, where the incoming input is highly improbable (deviant) based on the probability of the frequent events (standard). That is, the function of the “vMMN-generating system” is to update our predictive model of the world by means of prediction errors and infer the likely causes of the sensory inputs. *We interpret the vMMN as a prediction error signal to visual input that does not match probabilistic representations of the predicted (external causes of) input.* Unpredicted events carry a lot of information and can be important to survival. Thus, a further role that has been attributed to the mismatch signal is a trigger function for attention allocation (Nyman et al., 1990; Deouell, 2007). Attention is thought to increase precision of sensory signals (e.g., Feldman and Friston, 2010; Kok et al., 2012; Adams et al., 2013) and can deploy decision making and executive mechanisms.

The logic of the MMN studies rests on the usually hidden and rarely-studied process during which repetition of an event leads to the formation of a prediction pertaining to the probability of a given “feature” or “event” to occur. Such predictions in the MMN research are often referred to as “regularities” extracted from the stimulus stream (Winkler, 2007) and its presence is usually demonstrated indirectly by showing that stimuli that deviate from the frequent stimuli evoke a differential (mismatch) response. Most studies emphasize only one obviously beneficial aspect of automatic mismatch processes, namely the automatic registration of unpredicted changes in the environment, which has been suggested to trigger an attention orienting response (e.g., Kimura et al., 2008b). However, the other side of this coin is perhaps as much as important, namely the extraction and representation of the regular features, i.e., the formation of predictions (for a similar notion in auditory stream segregation see Schröger et al., 2014).

The extraction of the “common nominator” across repeating events leads to the representation of their invariant feature, which is the regularity itself. From this point of view the automatic build-up of a prediction corresponds to the process of *implicit category formation*, in a sense that a common feature which characterizes successive events has become active as an *ad-hoc* automatic “perceptual filter.” Thus, visual MMN seems to be suitable for studying whether a given visual “feature” is represented as an implicit category which serves as a basis for automatic discrimination processes and enables detection of remarkable/significant changes based on statistical characteristics of the environment. In summary, the vMMN is a universal tool which can be used to study automatic sensory discrimination and implicit (category) learning, i.e., a wide aspect of cognitive functions relying on visual information.

### Memory mismatch and refractoriness

Repetition of events lead to a response attenuation, a phenomenon often referred to as repetition suppression, stimulus-specific adaptation (SSA), habituation, refractoriness, or neural fatigue (Grill-Spector et al., 2006). Traditionally, amplitude decrease of ERP components over repetitions has been attributed to the decreased responsiveness of neurons for repeated input (Näätänen and Picton, 1987; May and Tiitinen, 2010). According to the “refractoriness” or “fatigue” model, in oddball sequences, neurons responding to the specific characteristics of the standard stimulus might acquire the refractory state, while the deviant stimulates “fresh” neural populations. Consequently, the amplitude of the exogenous (or obligatory sensory) ERP components evoked by the deviant will be larger than that of the standard. Such amplitude difference can be considered as a basic physiological phenomenon, without any cognitive functional significance. Alternatively, decreased activity can be considered as a manifestation of an active memory representation, established by the previous stimulation. The “predictive coding” account went a step further, suggesting that repetition suppression depends on the probability structure of the environment (see e.g., Summerfield et al., 2011) and involves an active process which generates models of the causes of the sensory input. These generative models can be thought of as hierarchical memory representations of stimulus characteristics, equivalent to predictive perceptual object representations (Winkler and Czigler, 2012). A stimulus that does not match this representation elicits a “mismatch process.” This process is manifested as an ERP component (MMN/vMMN). It is worth noting here that unpredicted omissions of attended (Bullock et al., 1994) and unattended (Czigler et al., 2006b) visual stimuli also evoke distinct ERP components which are difficult to account for based on the “fatigue” model, since there is no physical stimulus presented to activate “fresh” neural populations, although it is not known to what extent these components can be attributed to violated predictions and attentional effects. However, after more than three decades of research on MMN, the relationship of the “fatigue model” and “memory mismatch” (including the predictive coding account) has remained an unsettled issue (e.g., Näätänen et al., 2005; Garrido et al., 2009; May and Tiitinen, 2010; Wacongne et al., 2011, 2012; Todorovic and de Lange, 2012).

**Figure 1:**
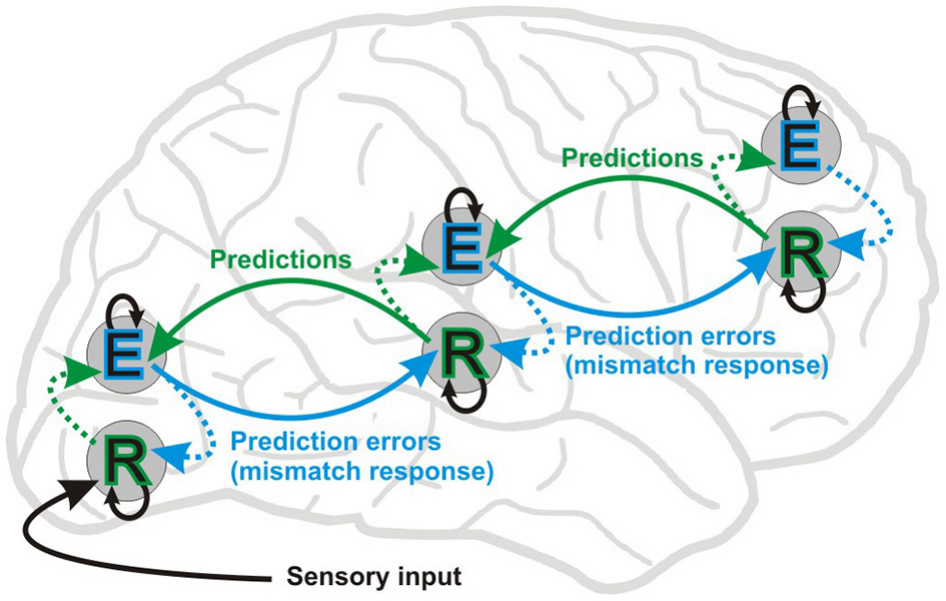
**Simplified scheme of the hierarchical predictive coding framework (Friston, 2005, 2008, 2010)**. The figure shows message passing between two putative neuronal populations: error units (E) and representation units (R). In this framework, bottom-up forward connections convey prediction errors (MMN or mismatch response) and top-down backward connections carry predictions, which explain away prediction errors (repetition suppression). Representation units residing in deep layers of cortical columns are thought to code the causes of sensory inputs. Representation units receive input from error coding units (E) in superficial layers in the same level (dotted lines) and lower hierarchical levels, and also from lateral connections at the same level (not shown). Lateral interactions between R and E units are proposed to select and sharpen R units, which in turn encode the causes of a given sensory inputs. Error units residing in superficial layers of cortical columns receive input from representation units in the same level and the level above. Inhibitory intrinsic connections are depicted by means of black arrows above and below E and R units, respectively. Perception depends upon a set of prior expectations, i.e., regularities extracted from earlier sensory events. Environmental statistical regularities are transformed into predictions about current sensory signals via the interaction of E and R populations. In MMN experiments using scalp EEG recordings the deviant ERP is contrasted to the standard ERP and components of their difference are commonly interpreted as manifestation of a prediction error signal. On the other hand, electrophysiological studies involving repetition suppression, i.e., the decrease in response amplitude over multiple presentations, provide only indirect evidence for the existence of putative representation units. That said, a recent functional magnetic resonance imaging (fMRI) study (de Gardelle et al., 2013) provides initial evidence for units coding perceptual predictions. Nevertheless, the hierarchical predictive coding framework elegantly accommodates the “fatigue model” and “memory mismatch” account of the visual and auditory mismatch negativity.

MMN/vMMN (or MMR) can be defined in at least two ways. In a broader and functional sense it is the ERP correlate of an automatic comparative process where the observed stimulus is different from perceptual memory representations of environmental regularities activated by recent external events. According to this definition, stimulus-specific response decrements to repeating events can be considered as a mechanism of memory match, and increased ERP amplitude to rare deviant events as a correlate of memory update. This is in line with the hierarchical predictive coding framework, where updating memory happens via a mismatch processes, i.e., prediction error responses update the models about external causes of the observed input (see Figure 1). The other definition is more restricted: “genuine” MMN/vMMN is the deviant-minus-standard differential activity, unless the difference is due to modulation by attention or refractoriness (passive amplitude reduction) of a negative ERP component, i.e., N1 (for the visual modality see e.g., Kimura, 2012). Separating “genuine” mismatch from activity due to passive amplitude reduction is important. If there is more than one process underlying stimulus–specific response decrements to repeating events, it is important to isolate these different kinds of activity and identify their potentially different contributions or functional roles.

The neurophysiological processes underlying regularity extraction, i.e., the formation of a predictive representation of stimulus features is not fully understood yet. A modeling study of the auditory MMN showed that experience-dependent plasticity can be explained by changes in the synaptic efficacy of extrinsic and intrinsic connections of sources generating the MMN (Garrido et al., 2008). Perceptual learning, caused by stimulus repetition, has been suggested to be brought about by changes in intrinsic and extrinsic neural connectivity corresponding to adaptation and prediction updating (model adjustment) processes, respectively (Garrido et al., 2009). Thus, reduction in response amplitude to repeated events is thought to be brought about by fast changes in synaptic connections (Baldeweg, 2006, 2007; Garrido et al., 2009) within and between hierarchical levels of neural elements which represent predictions based on previous events and generate MMRs (prediction errors) when deviation from prediction occurs (Friston, 2005). Figure 1 shows a simplified diagram of connections through which information flows between different layers of cortical columns at different hierarchical levels based on known functional anatomy (Zeki and Shipp, 1988; Douglas and Martin, 2004; Bastos et al., 2012).

According to this view (Friston, 2005, 2008, 2010), prediction errors flow bottom-up and update predictions at higher levels, whereas top-down modulations mediate predictions by “explaining away” (reduce) prediction errors at lower levels, forming hierarchical non-linear loops. Predictive coding theories of perception postulate that our internal model of probable causes of sensory events (i.e., reality) consists of a set of such loops (Winkler and Czigler, 2012) being supported by the complex hierarchical organization of brain networks (Kiebel et al., 2008; Wang, 2010; Arnal and Giraud, 2012).

#### Commonly used experimental protocols and procedures to elicit vMMN

**Figure 2:**
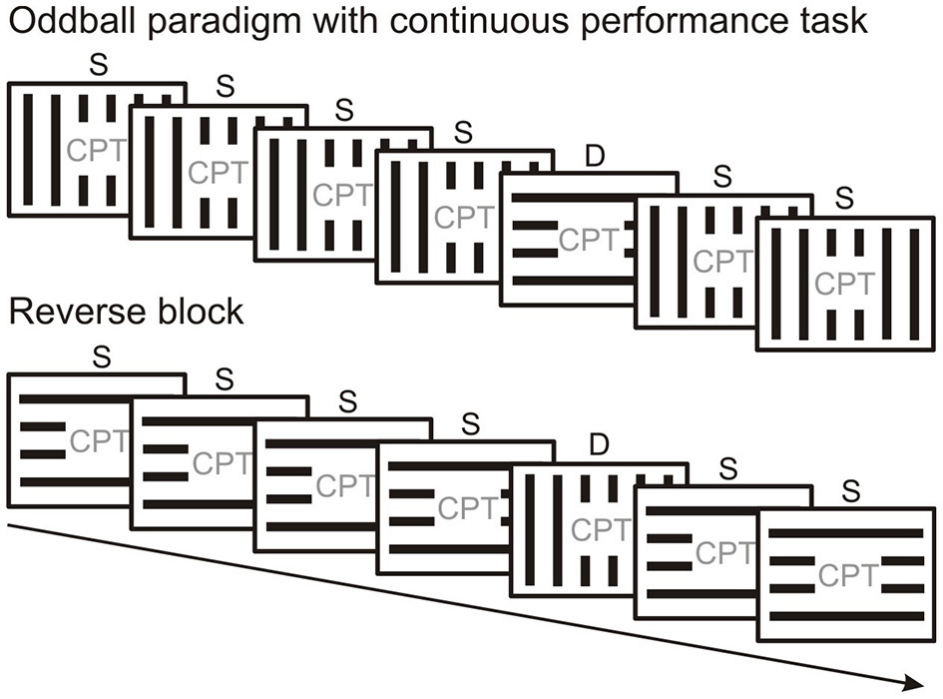
**Peripherally presented oddball stimulus sequence with a centrally presented continuous performance task (CPT)**. Standard (S) and deviant stimuli (D) are swapped across experimental blocks. MMN is calculated as the difference between original standard and deviant from the reversed condition (or vice versa) as they are physically identical.

There are mainly two kinds of protocols used to study vMMN. Since the vMMN is elicited by events which violate a probability-based regularity, these protocols systematically vary the probability of different stimulus types. Frequently used is the “oddball” paradigm, where the same type of stimulus is presented frequently, interspersed with a rare different stimulus which is sometimes referred to as “oddball.” There are essentially two types of oddball paradigm. In the “active oddball,” where the rare stimulus is usually task-relevant and attended, the rare stimulus is termed “target,” and is used to elicit P3b/P300 and other attention-related components. In vMMN experiments the “passive oddball” is used (Figure 2), where the stimulus stream which is used to build up automatic predictions is unattended, the rare stimulus (or stimulus feature) is task-irrelevant and is termed “deviant,” emphasizing its difference from the frequent “standard.”

Stimuli in every sensory modality elicit exogenous (obligatory) ERP components. The amplitude and latency of these components depends on the physical characteristics of the stimuli (e.g., luminance, contrast, or spatial frequency) and stimulus conditions (e.g., the time interval between successive stimuli). If deviant and standard stimulus categories are not equated appropriately, then the deviant minus standard difference wave is a summated activity of mismatch-related processes and brain electric activity in response to other different stimulus characteristics. This latter activity is not elicited by the violation of the probability-based rule established by the pattern of the stimulus sequence, and it might confound the vMMN. It is not known how variability of stimulus features—other than on which the probability-based rule rests—affects the mismatch generation process. Therefore, in experiments where vMMN is used as a tool to address a specific question of automatic information processing in the brain, it is advisable to make sure that *different stimulus types differ only in that feature which carries the distinctive information*^2^, i.e., which defines the standard vs. deviant stimulus categories.

Figure 2 illustrates the oddball paradigm. In the traditional passive oddball paradigm the standard and deviant stimuli differ in their (i) physical properties and (ii) probability. Correspondingly, (i) different (although potentially overlapping) neuronal pools will respond to the standard and deviant and (ii) their level of adaptation will differ. A frequently applied solution to control for potential ERP differences arising due to differences in physical stimulus properties involves changing the probabilities of standard and deviant stimuli across experimental blocks (e.g., Stefanics and Czigler, 2012; Stefanics et al., 2012; Csukly et al., 2013). Running a “reverse block” generates data that allows comparison of ERPs to physically identical stimuli which served both as standard and deviant in different experimental blocks and thus eliminates one of the potential confounds inherent in the design of an oddball paradigm. Although reverse blocks for oddball series offer stimulus conditions which allow control for physical differences between standard and deviant, they do not control for repetition effects arising from the difference in the presentation rate between standard and deviant.

**Figure 3:**
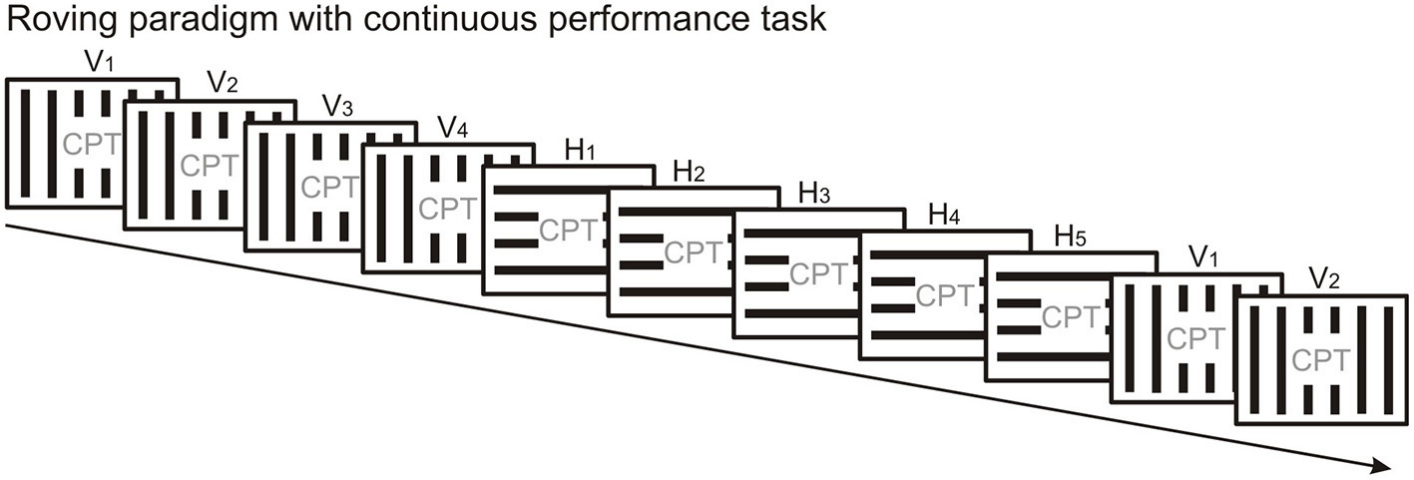
**The roving standard paradigm presents the physically different stimuli with equal overall probability**. Thus, the standard and deviant stimulus categories are not defined by their overall but their local probabilities and they change with the stimulus position in the stream. Here microsequences of vertical (V) and horizontal (H) gratings alternate. The first stimulus in a microsequence is a “deviant” since it violates the regularity established during the previous microsequence. The inherent design of the roving paradigm allows studying the time course of repetition effects. A continuous performance task is presented in the center of the screen to engage the participant's attention.

The less frequently used protocol to elicit vMMN is the “roving standard” paradigm (Figure 3), where the first stimulus in the train can be considered as “deviant” which over several repetitions becomes the “standard.” An advantage of the “roving standard” compared to the oddball paradigm is that it allows studying repetition effects following stimulus change, i.e., the time course of response decrement over repetitions. The roving paradigm has only been used in few vMMN studies so far (Czigler and Pató, 2009; Sulykos et al., 2013), and these studies did not take advantage of the roving protocol to study repetition effects. In terms of experiment duration, running a roving standard paradigm should take less time than running an oddball sequence and its “reverse” control condition, provided that the deviant/standard ratio is the same in both paradigms. Thus, the roving paradigm is less demanding for participants, which might be particularly important in case of children and patient populations.

#### Exogenous ERP components in the vMMN range

In case of interest in mismatch-related processes beyond the stimulus-specific refractoriness, it is important to separate the probability effects on the exogenous components and the putative additional activity. ERP components are often classified as exo- or endogenous (Donchin et al., 1978; Näätänen, 1992; Koelsch, 2012). External stimuli are necessary and sufficient to elicit exogenous components and they are main determinants of the characteristics of exogenous components, whereas external stimuli are not necessary to elicit endogenous components which are dependent on factors such as attention and intention. Compared with visual ERPs, the succession and scalp distribution of exogenous auditory components (N1, P1, and N2) is remarkably stable. Most importantly, in the present context, reliable auditory N1 emerges over the anterior scalp within the 70–160 ms latency range. The auditory N1 consists of several sub-components with different latencies, scalp distributions and refractoriness characteristics (Budd et al., 1998). However, the N1 is treated as a single component in the majority of MMN studies. To claim that at least a part of the deviant-related negativity in vision is due to refractoriness, it is necessary to identify functionally similar exogenous component(s). In fact, the N1 visual component is present in many visual ERP studies, and traditionally, this component is treated as the analog of the auditory N1. However, the component structure of exogenous visual potentials is highly variable. Furthermore, the set of exogenous components in vision is more complex. The onset of visual stimuli might elicit luminance and pattern-specific ERP components. The latency and polarity of these components depend on the stimulated part of the visual field (Jeffreys and Axford, 1972; Di Russo et al., 2002). The interaction of the luminance and pattern-related activity adds further variability to the scalp-recorded waveform. The polarity and amplitude of scalp-recorded ERPs depend on the spatial orientation of their underlying (dipolar) sources (Di Russo et al., 2002, 2003), which is in turn defined by the particular folding structure of the cortical generator area and its relative position to the active and reference electrodes. Taking into account the spatial extent of visual brain areas and their complex folding structure, it is easily conceivable that some deviant minus standard difference waves will show not only deviant-related negativity but also deviant-related positivity at some posterior sites. Accordingly, although several vMMN studies indicate that in the vMMN latency range the event-related activity is dominantly negative over the posterior locations (over the visual brain areas), in other studies, no characteristic negativities have been recorded.

In the auditory modality, the repetition-related N1 decrement within a stimulus sequence occurs mostly between the first and second stimulus presentation, without hardly any decrement with further stimulus repetitions (Budd et al., 1998), suggesting that refractoriness is the main reason underlying the N1 amplitude decrement. In this study, ERP amplitudes to the first and subsequent stimuli were investigated after a long silent period. Such a decrement results from the combined effect of non-specific factors and factors specific to the repetition of particular stimulus features (stimulus specific refractoriness). In a recent electrocorticography (ECoG) study using an auditory paired stimulus paradigm numerous cortical regions were found to generate remarkable N1 responses, and about half of them, including frontal, orbito-frontal, cingular, parietal, and temporal areas exhibited significant repetition suppression effects (Boutros et al., 2011). This finding suggests that N1 amplitude suppression might result mainly from active processes, and not only from passive refractoriness. Importantly, the difference in the topography of the initial response and the repetition effect suggests that these two functions are supported by distinct neural circuitries. Refractoriness changes as a function of the duration of stimulus onset asynchrony (SOA); therefore, using longer intervals between consecutive stimuli, a smaller amplitude difference is expected. As for the visual modality, according to recent studies, the SOA effects on posterior visual ERP components are not particularly large. Coch et al. (2005) observed no amplitude increase in the N1 range between 450 and 650 ms SOA, while the preceding positivity was larger at the longer SOA value.

In some studies, the application of repeated stimuli after a stimulus change (AAABAA) did not elicit decreased exogenous activity. In an oddball sequence, Kimura et al. (2010d) compared the ERPs of the first and second standard after a deviant in a task with orientation deviancy. The orientation of stimulus bars was task-irrelevant; participants had to respond if the bars had round but not square edges. In this study, the peak of the negative component of the first and second standard after a deviant at ~150 ms was not different; the ERPs of the first and second deviants diverged somewhat later, at ~170 ms. Furthermore, there was no difference between the ERPs of the second standard and the average of the standard-related ERPs. Czigler et al. (2006a) presented colored checkerboard stimuli in a regular AABBAABB order (A and B corresponding to red and green), with 350 ms SOA, where the deviant was an unpredicted repetition of a color, e.g., BBAAA). According to the “refractoriness” model, the repeating predicted stimulus (e.g., AA) is expected to elicit smaller exogenous activity. However, such stimuli elicited larger posterior negativity than the regular change (e.g., AB). Moreover, Stefanics et al. (2011) recorded ERPs in a sequence of paired stimuli with equal probability of within-pair color change or color differences. The between- and within-pair SOA was 800 and 300 ms, respectively. In this study, the stimulus change and stimulus repetition elicited almost identical ERPs. Findings of a recent fMRI study might resolve these seemingly controversial results. de Gardelle et al. (2013) presented subjects with repeating face stimuli and found that distinct patches of face-responsive extrastriate region showed simultaneously repetition enhancement and suppression responses to repetitions. This finding is consistent with the predictive coding account which posits representation (prediction) coding units enhance their activity and error coding units show decreased activity over repetitions.

To demonstrate the relationship between exogenous activity and the deviant minus standard difference potentials, here we survey studies which used deviant stimulus orientation to elicit vMMN. This type of deviant has been applied in several studies in various laboratories, and it was also used in studies that attempted to eliminate refractoriness effects using the so called “equal probability control” condition. Kimura et al. (2009) presented single gray bars in the center of a dark screen (stimulus with luminance increase). The stimuli elicited a posterior positivity with ~100 ms latency (P1), followed by negativity with ~150 ms latency (N1). Astikainen et al. (2008) presented a single dark bar in the center of a gray background (stimulus with luminance decrease). In this study, a large posterior positivity emerged with ~140 ms latency, and the subsequent negativity with ~210 ms peak latency. Kimura et al. (2009) showed that the deviant minus standard difference emerged as a parieto-occipital negativity in the 100–250 ms range, while Astikainen et al. (2008) showed negativity in the 185–205 ms range. Czigler and Sulykos (2010) presented a texture of colored oblique lines in a dark field. The latency of the posterior negativity was ~130 ms, followed by positivity with ~250 ms latency. Deviant-related negativity appeared in the 130–190 ms interval, with peak latency of ~160 ms, i.e., the difference potential peaked later than the exogenous negativity. Sulykos and Czigler (2011) presented a set of gray-scaled Gabor-patches in a dark stimulus field, either to the lower or upper half of the visual field. The lower half-field stimulation elicited a posterior positive-negative-positive sequence of potentials with ~100, ~150, and ~240 ms peak latencies, respectively, whereas the polarity of the components was reversed in the upper half-field stimulation (~100, ~170, and ~260 ms peak latencies, respectively). The deviant minus standard difference potential also showed polarity reversal depending on which hemifield was stimulated, and its peak latency was 130 ms at lower half-field stimulation and 132 ms at upper half-field stimulation, i.e., the deviant-related activity appeared earlier than components in the “N1” or “inverted N1” range. Takács et al. (2013) presented a set of task-irrelevant Gábor-patches with deviant and standard orientations to the whole visual field while participants performed a tracking task presented in the center of the visual field. Over the occipital scalp Gabor-patches elicited a positive-negative-positive complex with ~90, ~110, and 240 ms peak latencies, respectively. At occipito-temporal locations, a further negativity emerged with 170 ms peak latency. Deviant-related negativities emerged in the 120–140 and ~200–230 ms intervals, i.e., outside the ranges of the exogenous negativities.

Some of the above studies (Astikainen et al., 2008; Czigler and Sulykos, 2010) showed that the posterior negative difference potential appeared in the range of a positive ERP component. Similar examples were observed in studies with other deviant features (see e.g., Czigler et al., 2002; Liu and Shi, 2008; and Stefanics et al., 2011 for color; Kremláček et al., 2006 and Pazo-Alvarez et al., 2004b for motion direction; Maekawa et al., 2005 for shape/spatial frequency). However, none of these studies reported “mismatch positivity” at posterior sites, i.e., a potentially refractoriness-related effect appearing as a positive difference potential. To our knowledge, no argument has been presented for the exclusive sensitivity to refractoriness of posterior negative ERP components and the lack of refractoriness in the case of positive components. Nevertheless, it is worth noting that positive components of the deviant minus standard waveforms have been observed at central (Stefanics et al., 2012; Csukly et al., 2013) and frontal (Stefanics and Czigler, 2012) sites, evoked by deviant facial emotions and hand laterality, respectively, which correlated with behavioral measures.

#### The equal probability control for repetition effects

**Figure 4:**
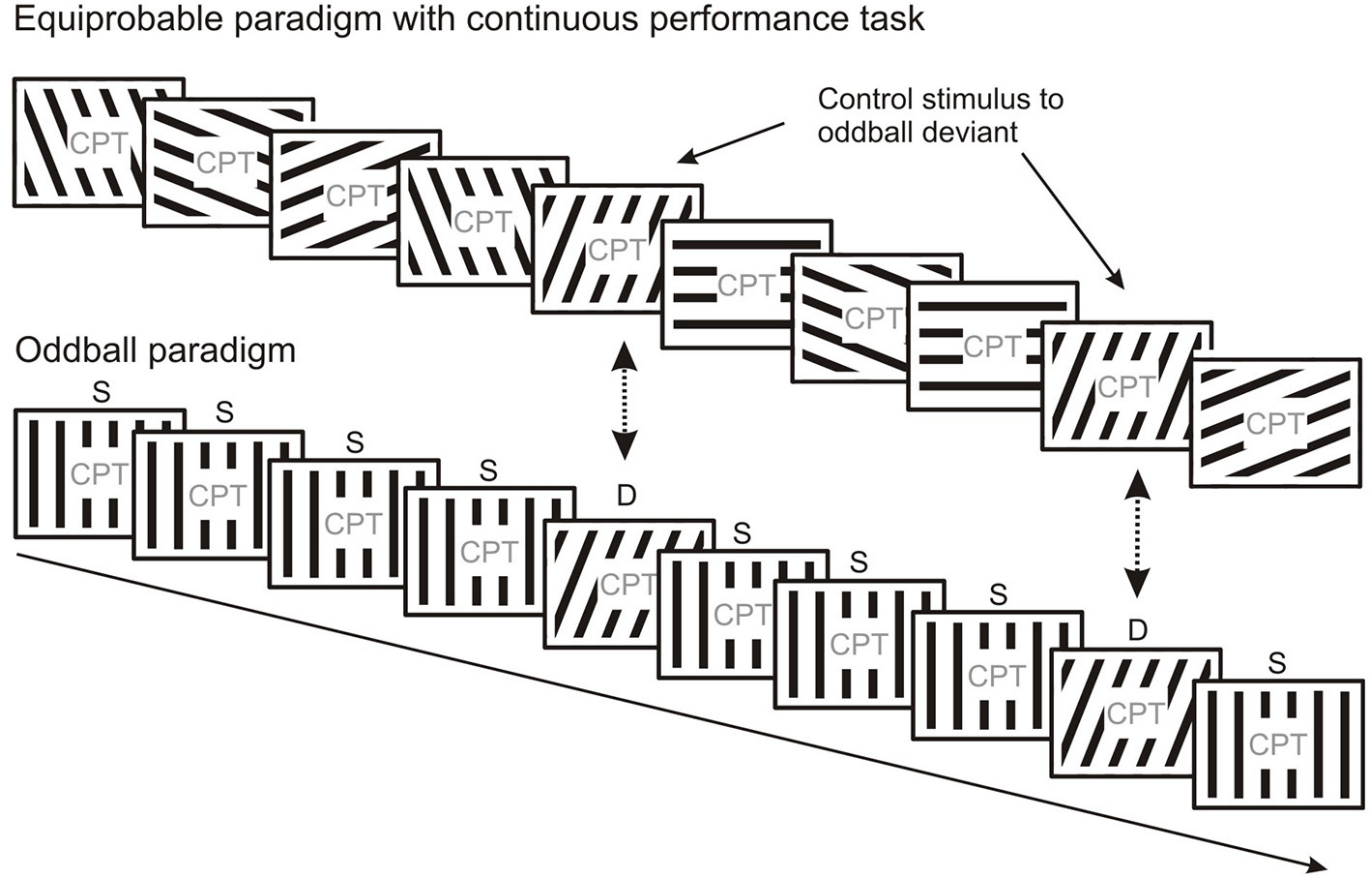
**The equiprobable paradigm can be used as a control for oddball paradigms**. In the equiprobable paradigm each stimulus type occurs with the same probability, i.e., no frequent “standard” and rare “deviant” stimulus categories are present. Responses evoked by stimuli physically identical to those evoked by deviants in the oddball block can be compared. The equiprobable paradigm is thought to control for refractoriness effects induced by frequent repetitions of the standard in the oddball paradigm.

Schröger and Wolff (1996) and Jacobsen and Schröger (2001) suggested the elegant method of equal probability control to deal with repetition effects due to refractoriness assumed to be present in the deviant minus standard activity obtained in oddball paradigms. This method allows comparison of ERPs elicited by the deviant of the oddball sequence to the ERPs elicited by physically identical stimuli from a sequence without one particular frequent (standard) stimulus. In the equal probability control condition (Figure 4) stimuli with a structured set of parameters are presented where the mean difference between consecutive stimuli is equal to or larger than the difference between the deviant and standard used in the oddball sequence, furthermore stimuli identical to the oddball deviants have the same probability as the deviants. Activity considered as “genuine” MMN (i.e., MMN without stimulus specific refractoriness effects superimposed) emerges when the oddball deviant elicits larger negativity than the control stimuli. It should be noted, that the equiprobable control can be considered as a sequence of deviants where each stimulus violates the expectation based on the previous stimulus, i.e., that a given stimulus would repeat. Therefore, the ERP to the equiprobable control stimulus probably contains weaker prediction error responses than those to oddball deviants since there is less sensory evidence available for every external event in the equiprobable control condition due to the lack of sequential stimulus repetitions. From a probabilistic point of view, the “genuine” vMMN to the oddball deviant reflects a prediction error to events which violates expectations based on stronger sensory evidence provided by frequent standard stimuli.

Studies employing changes in line orientation have illustrated the relationships between deviant-related negativity and exogenous components using the equal probability control. These studies (Astikainen et al., 2008; Kimura et al., 2009) were discussed above in the context of the relationship between the exogenous and deviant-related negativities. Kimura et al. (2009) showed that the equal probability control efficiently removed the early part of the deviant-related negativity of oddball sequences. As a result, “genuine vMMN” appeared in the 200–250 ms range. Astikainen et al. (2008) showed that the deviant minus equal probability control difference resulted in a less broad distribution of the difference potential over posterior locations, but the latencies (185–205 ms) were identical in the deviant minus standard and deviant minus control differences. In a recent study, Kimura and Takeda (2013) presented a set of gray bars on a dark field and recorded exogenous activity at parieto-occipital locations with ~180 ms peak latency for the deviants and controls, whereas the standard elicited no N1-related negativity. The deviant minus standard difference potential resulted in long-lasting bilateral negativity within the 120–250 ms range. The amplitude of the deviant minus control difference (“genuine vMMN”) was much smaller and restricted to the right side indicating that the equal probability control dissociated the effects of exogenous components and an additional posterior negativity.

Schröger (1997) and Ruhnau et al. (2012) argued that the equal probability control overestimated the effect of refractoriness. This is because oddball is a regular sequence, whereas the equal probability control is an irregular one. Therefore, an “irregularity effect” might add to the lack of stimulus repetition. They proposed a sequence called cascadic control. In this sequence stimuli with various characteristics are ordered in upward-downward sub-sequences, preserving regularity, and stimulus variability (and avoiding stimulus repetition). In this study the random equal probability control elicited larger N1 than the oddball deviant and cascadic equal probability control suggesting that the random equal probability control might overestimate frequency-specific repetition effects^3^. File et al. (in preparation) compared vMMN of the traditional oddball paradigm, the equal probability control and the cascadic control. The deviant set of bar pattern had different orientation than the standard. Both the equal probability and the cascadic control eliminated the deviant-related effect in the 120–160 ms interval.

In addition to studies on orientation deviancy, equal probability control was introduced in three other studies. Czigler et al. (2002) investigated color-related deviance and obtained similar posterior negativity in the deviant minus control and deviant minus standard difference potentials. In this study, the average distance between the various colors of the control condition was not necessarily larger than the distance between the standard and deviant; therefore, the control condition did not guarantee non-refractory ERPs. However, in this study, the latency of the exogenous posterior negativity was 100 ms, whereas deviant-related activities emerged later, in the 128–142 ms range, where the exogenous activity was positive. In this study, the standard elicited the largest exogenous negativity. Li et al. (2012) used equal probability control to study emotion-related vMMN. Facial emotions are categorically different; therefore the magnitude of the distance within the oddball and control sequences is meaningless. In the oddball condition, the standard face was neutral and the deviant face was sad, whereas in the control conditions, three additional emotions were added to the sequence. Both the deviant minus standard and the deviant minus control difference potentials were negative within a long range (100–400 ms) over the occipito-temporal regions. In the latency range of the exogenous negative component, the negative difference was smaller (but present) in the deviant minus control difference potential, suggesting the contribution of refractoriness for the standard face of the oddball sequence. Recently, Astikainen et al. (2013) also used equal probability control to study emotion-related vMMN. In the oddball sequence rare fearful and happy faces were presented among frequent neutral faces, whereas in the equal probability condition all three expressions were presented with the same probability. The independent component analysis showed that the deviant minus standard differential negativity at ~130 ms was larger at right posterior sites than the deviant minus control difference potential, indicating that a portion of the deviant minus standard negativity could be explained by repetition effects.

In summary, the results of equal probability control suggest that stimulus-specific repetition effects might contribute to the increased negativity to the deviant stimulus. Whether these effects reflect basic neurophysiological processes without functional significance in perceptual learning is still an open issue, although it is unlikely to be the case (cf. predictive coding theories). However, majority of the studies indicated the emergence of a posterior negativity, which cannot be attributed to the refractoriness of the endogenous components. Furthermore, considering the results of these studies and the results showing that deviant-related negativity might precede or follow negative exogenous components, there is no unequivocal evidence that the additional negativity (genuine vMMN) emerges later than exogenous activity. Applying equal probability control in future studies to obtain results allowing generalization to other features than line orientation is recommended.

#### Other methods to control repetition effects (refractoriness)

To investigate the effects of repetition, it is possible to compare the ERPs of the deviant of the oddball sequence to the ERPs elicited by identical stimuli from sequences without the standard stimuli (“lonely deviant”). If memory representation of the standard is necessary for the emergence of the deviant-related activity, an additional negativity is expected in the deviant minus standard difference potential. Without such additional activity, the similarity of the negative ERP component (similar latency and scalp distribution but larger amplitude for the lonely deviant) supports a refractoriness effect. Kenemans et al. (2003) using changes in special frequency of grating stimuli found a posterior negativity with similar latency and scalp distribution for the “lonely deviant” and in the deviant minus standard difference potential, supporting the refractoriness account. Due to the larger interval between the stimuli (decreased non-specific refractoriness); the negativity to the lonely deviant was larger. However, in a similar study, Astikainen et al. (2004) did not obtain a similar increased negativity using tilted bars as the standard deviant and “lonely deviant.” Berti and Schröger (2006) investigated the distracting effects of task-irrelevant stimuli on duration discrimination tasks. In an oddball condition in the standard trials, the stimuli (triangles) were presented to the center of a screen, but infrequent stimuli were presented at either of two eccentric positions. In a control condition, the probability of stimulation in the three possible positions was equal, and in another control condition, the probability of the central position was equal to the sum of the probabilities of the eccentric position. Accordingly, in the oddball condition, the standard acquired a probability-based regularity, whereas no such regularity was present in the equal probability, 50% standard, and 25–25% deviant conditions. Deviant-related posterior negativity of ~220 ms latency (N2p according to the authors' terminology) appeared only in the oddball condition. This negativity might be associated with the vMMN, and the results show that rareness itself is not enough to elicit this component.

Indirect support for “refractoriness” in the N1 latency range was provided by Kimura et al. (2008a,2010b). Higher stimulus intensity is expected to increase response amplitude, i.e., a deviant with higher luminance should elicit larger N1 due to the additional exogenous activity, which in turn might contribute to the deviant minus standard difference. In these studies larger negativity appeared for deviants with higher luminance, but not for deviants with less intensity. Stagg et al. (2004) also compared the effects of brighter and darker deviants. In their study vertical bars were presented to the upper and lower half of the visual field, and both luminance and the deviancy-related effects appeared after the N1 negativity. While both the bright and dark bars elicited similar deviant-related negativity in the 210–400 ms range (comparison between identical stimuli as deviant vs. standard), the bright stimuli elicited larger negativity (comparison between the bright and dark stimuli). Therefore, in this study, the effect of physical difference and the deviant-related activity was additive.

In summary, deviant-related negativity cannot be fully explained on the basis of stimulus-specific refractoriness. At the same time, the contribution of repetition effects and stimulus-specific refractoriness cannot be ruled out.

#### Stimulus-specific adaptation and refractoriness

The effect of SSA of the oddball sequences can be viewed in the context of adaptation studies, where the adaptor stimulus is presented first, sometimes for a longer time, followed by a probe stimulus. The effect of an adaptor is stimulus-specific, both at the level of behavioral performance and ERP activity (e.g., Webster and MacLin, 1999; Eimer et al., 2010; Kloth et al., 2010; Eimer, 2011; Zimmer and Kovács, 2011). The adaptation effect is widely considered as an index of an acquired specific memory representation. There is apparently a discrepancy in the interpretation of repetition effects between fields using the adaptation method and the oddball task, as in the former field repetition-related changes are thought to reflect memory formation (e.g., Desimone, 1996; Ringo, 1996), whereas in the latter field a decrease in response amplitude is often considered as an irrelevant neurophysiological effect reflecting neuronal “fatigue” or “refractoriness” (e.g., Maess et al., 2007).

In functional MRI, using adaptation effects (repetition suppression) is a standard mapping tool to identify brain regions associated with different stages of stimulus-processing and to investigate memory representation (e.g., Henson, 2003; Grill-Spector et al., 2006; Kovács et al., 2013), even though the relationship between repetition suppression and repetition enhancement is a more complex issue (Segaert et al., 2013). For example, Park et al. (2007) observed decreased activity in brain areas sensitive to visual scenes if a scene was preceded by a similar scene, but from a narrower view. This difference was attributed to an effect called boundary extension (Czigler et al., 2013), and interpreted as a proof of the illusory memory representation of scenes represented together with a broader background.

Mismatch negativity has a potential analog in the stimulus repetition effects measured with single-cell recording in a variety of species including mice, cats, rats, owls and primates. SSA is the closest known single-neuron phenomenon of MMN (for reviews see Nelken and Ulanovsky, 2007; Escera and Malmierca, 2014). SSA is a non-trivial effect, since use dependence (refractoriness or fatigue) cannot account for SSA (Nelken and Ulanovsky, 2007). SSA and the auditory MMN show remarkable similarities. The magnitudes of SSA and MMN are both negatively correlated with the probability of the deviants but positively correlated with the difference between standard and deviant. However, an important difference is that the earlier timing of SSA relative to MMN, which led Nelken and Ulanovsky (2007) to suggest that SSA is a correlate of change detection in the primary auditory cortex upstream of MMN, and that MMN itself is a compound response of primary and higher-level cortical areas with longer response latencies. Beside in cortical neurons, SSA has been observed in subcortical structures, such as the superior colliculus and thalamus as well, supporting the notion of a hierarchically organized changed detection system (Grimm and Escera, 2012; Escera and Malmierca, 2014) which is in line with the hierarchical predictive coding framework.

Although the exact mechanisms and neurophysiological effects of stimulus specific adaptation in the visual system are not fully understood yet, at least three mechanisms have been identified, including somatic afterhyperpolarization, synaptic (network) mechanisms, and synaptic depression due to the depletion of vesicles from the presynaptic terminal (for a review, see Kohn, 2007). It is important to note that only one of the three contributing mechanisms of adaptation, namely depletion of neurotransmitter vesicles is in line with the interpretation of repetition effects according to the passive “refractoriness” model. Furthermore, SSA has more complex properties than is usually assumed from neural “refractoriness” in human electrophysiology (Nelken, 2012; Nelken et al., 2013). However, it is relatively unknown whether mechanisms underlying the repetition-related amplitude reduction and the increased response to “unpredicted” events interact. In cognitive terms the processes supported by these mechanisms correspond to the build-up of predictions (internal model of the environment), and change detection (model update). Human ECoG recordings indicate that not every brain site that responds to repeated tones show repetition suppression (Boutros et al., 2011), thus it is plausible that the initial response to the unpredicted stimuli and repetition suppression are two linked, but separate, functions.

At this stage, two outstanding issues can be pointed out. First, is there a relationship or interaction between the processes underlying the repetition-related amplitude decrement for the standard (adaptation, refractoriness, or repetition suppression) and the increased activity to the deviant (“genuine” mismatch negativity)? According to the hierarchical predictive coding framework (Friston, 2005, 2008, 2010) the two processes are mutually linked and influence each other. Neurophysiological (Ulanovsky et al., 2003) and ERP findings (Boutros et al., 2011) as well as empirically based models (Garrido et al., 2009) argue for the contribution of “refractoriness” to the mismatch process. However, there is no direct empirical evidence in the vMMN literature for a link between the change of the exogenous components (as memory representation of the standard) and the detection of changing stimulation. Second, are there any other correlates (ERP or other) associated with vMMN-related memory representation (i.e., to-be-mismatched memory)? In the auditory modality, Haenschel et al. (2005) described a positive ERP component for stimulus repetition (repetition positivity, see also Costa-Faidella et al., 2011). Until recently, no visual analog of this component has been reported, although a recent fMRI study by de Gardelle et al. (2013) presented subjects with repeating face stimuli and found that distinct patches of face-responsive extrastriate region showed concurrent repetition enhancement and suppression to repeated stimuli. As previously mentioned, some studies have shown that vMMN was apparently independent of the “refractoriness” of exogenous activity. In these cases, we have no data concerning the memory acquisition and retention processes, and it is possible that these processes are different from those underlying the decreased amplitude of the exogenous components or repetition positivity.

### Visual MMN and attention

vMMN is thought to be a neural correlate of automatic perceptual processes. To identify components of the deviant minus standard difference potential as a vMMN, it is necessary to ensure that the eliciting stimuli remain outside the focus of attention. It is important to recognize that this issue has both theoretical and methodological significance. In the hierarchical predictive coding framework the major task of the perceptual system is to predict future events as precisely as possible (Muckli, 2010). Attention is thought to modulate the precision of prediction errors by altering the gain of error-units (Friston, 2005, 2010). Higher precision means less uncertainty of prediction errors. According to this hypothesis, attention increases the weight of error units processing certain features or events and controls their relative influence at different levels (c.f. Bowman et al., 2013). The momentary strength of top-down and bottom-up interactions is dynamic, with attentional processes being able to modulate the weight of prediction errors (Clark, 2013). Accordingly, recent functional MRI findings support such a predictive coding model where top-down predictions attenuate sensory signals while attention can reverse such effects (Kok et al., 2012). Apart from theoretical considerations, from a methodological point of view, task-relevant or otherwise attended stimuli elicit posterior negativities in comparable latencies (e.g., Harter and Guido, 1980; Czigler and Csibra, 1990; Kenemans et al., 1993; Torriente et al., 1999), that is attentional effects might easily confound MMRs. Therefore, careful control of attentional processes is necessary for the identification of posterior negativities as vMMN.

In the majority of auditory MMN studies, attention to the MMN-related stimuli is reduced by visual tasks. Experimental protocols often involve watching a silent movie or reading a book, and due to lack of behavioral indicators of attentional involvement, it is difficult to gauge to what extent attention might be involved in those studies. Nevertheless, the claim that auditory MMN can be elicited independent of attention is supported by studies showing a MMR in sleeping newborns (Stefanics et al., 2007, 2009; Háden et al., 2009), sleeping adults (Nashida et al., 2000; Atienza and Cantero, 2001; Sculthorpe et al., 2009), and comatose patients (Kane et al., 1993, 1996; Fischer et al., 1999). In the majority of vMMN studies, the concurrent tasks are also visual, because in the absence of other relevant visual events it is difficult to withdraw attention from visual stimuli. Vision is usually considered as the dominant sensory modality, at least at the pre-response level, where visual distractors cause more interference to auditory processing than vice versa (Chen and Zhou, 2013). Several different protocols have been used to keep the participants' attention engaged and away from the mismatch-evoking stimuli. Table 1 summarizes different approaches that have been used to reduce attention to the vMMN-related sequences. As a prototypical example, Winkler et al. (2005) instructed participants to detect infrequent stimulus changes of a central fixation cross, while mismatch-evoking stimuli were presented in the background. From time to time, the cross became wider or longer, which participants had to indicate with a button press. After the experiment participants were debriefed about the vMMN-related stimuli and the stimulus changes. According to their reports, they did not notice the regularity within the sequences. Czigler and Pató (2009) used a similar central task arrangement and debriefed participants in a detailed interview about their experiences. According to the answers, they were unaware of the changes within roving standard sequences. In spite of the lack of awareness, changes elicited posterior negativities. After an instruction that brought the changes within the sequence to the attention of participants both scalp distribution and latencies of the negativities were markedly different.

**Table 1:** **A number of tasks have been used in different studies to reduce attention to events evoking the vMMN**.

**Task**	**References**
Tracking	Heslenfeld, 2003; Yucel et al., 2007; Sulykos and Czigler, 2011; Kecskés-Kovács et al., 2013a; Takács et al., 2013
Deviant in attentional blink position	Berti, 2011
Central task, independent of the sequence of vMMN-related stimuli	Czigler et al., 2002, 2004, 2006a,b; Lorenzo-López et al., 2004; Pazo-Alvarez et al., 2004a,b; Besle et al., 2005, 2007; Winkler et al., 2005; Amenedo et al., 2007; Czigler and Pató, 2009; Flynn et al., 2009, Experiment 2; Kimura et al., 2010a; Müller et al., 2010, 2012; Urakawa et al., 2010a,b; Qiu et al., 2011; Stefanics et al., 2011, 2012; Stefanics and Czigler, 2012; Cléry et al., 2013a,b; Kecskés-Kovács et al., 2013b; Kimura and Takeda, 2013; Kremláček et al., 2013; Shi et al., 2013; van Rhijn et al., 2013; Kovács-Bálint et al., 2014; Si et al., 2014; Sulykos and Czigler, 2014
Central task with the standard and/or deviant of the vMMN-related stimuli	Kenemans et al., 2003, 2010; Kimura et al., 2006a, 2010b—“independent” condition; Grimm et al., 2009; Clifford et al., 2010; Mo et al., 2011; Cleary et al., 2013; Kuldkepp et al., 2013; Shtyrov et al., 2013; Stothart and Kazanina, 2013; Tang et al., 2013
Central task, within the sequence of vMMN-related stimuli	Tales et al., 1999, 2002, 2008, 2009; Stagg et al., 2004; Maekawa et al.*, 2005; Kimura et al., 2006b,2010c; Kremláček et al., 2006; Tales and Butler, 2006; Fonteneau and Davidoff, 2007; Hosák et al., 2008; Liu and Shi, 2008; Urban et al., 2008; Athanasopoulos et al., 2010; Chang et al., 2010, 2011; Froyen et al., 2010; Susac et al., 2010; Kimura, 2012; Files et al., 2013; Fujimura and Okanoya, 2013; Kreegipuu et al., 2013; Maekawa et al., 2013; Wang et al., 2013. ^*^Together with an auditory task.
Feature of the task-related stimuli	Fu et al., 2003; Berti and Schröger, 2006; Berti, 2009; Kimura et al., 2009, 2010d; Müller et al., 2013
Auditory task	Horimoto et al., 2002; Wei et al., 2002; Astikainen et al., 2004, 2008, 2013; Astikainen and Hietanen, 2009; Zhao and Li, 2006; Chen et al., 2010; Fisher et al., 2010; Khodanovich et al., 2010; Gayle et al., 2012; Tomio et al., 2012
Fixation, or target-related vMMN stimuli	Mazza et al., 2005; Flynn et al., 2009, Experiment 1; Ponton et al., 2009; Lyyra et al., 2010; Kogai et al., 2011

*Different approaches are listed according to their putative efficiency to engage the participant's attention in tasks that are irrelevant to the vMMN-evoking stimuli*.

Using an attentional blink paradigm, Berti (2011) investigated the potential involvement of attention in mismatch generation more directly. In this elegant experiment, irrelevant deviant stimuli (stimuli in deviant location) followed the target events at various lags in rapid serial visual presentation (RSVP) sequences. A robust result of attentional blink studies is that if the target is followed by another target stimulus within an interval of ~100–500 ms, the probability of detecting the second target decreases (e.g., Dux and Marois, 2009). Berti (2011) observed vMMN in the attentional blink interval, indicating that no attentional processing is needed for the emergence of this ERP component.

Continuous tasks, such as tracking and RSVP of sequences together with para-foveal or peripheral stimulation, seem to be the most stringent controls. A somewhat less strict method is the introduction of detection tasks at the fixation point together with presentation of the vMMN-related stimuli outside the fixation field. As for the ecological validity of this spatial arrangement of the stimuli, in everyday situations unattended but important events first occur outside the center of our visual field. In a perhaps more effective variant, the onset time of task-related (target) stimuli is independent of the appearance of vMMN-related stimuli; in the other version, the onset of the task-relevant stimuli coincides with that of the vMMN-related stimuli (and usually of the standards). Furthermore, reduction of attention to the vMMN-related stimuli is presumably weaker if the target stimuli are members of a sequence of vMMN-related events. This arrangement is similar to the three-stimulus oddball paradigm (Katayama and Polich, 1998, 1999). In some studies, vMMN-relevant stimulus features are present also in the task-relevant objects. A problem with this design is that studies on object-related attention have shown that irrelevant features of task-related stimuli cannot avoid attentional processing (e.g., Duncan, 1984).

A set of studies attempted to translate auditory MMN protocols by presenting the task-irrelevant visual stimuli together with the task-relevant auditory stimuli. To reduce the saliency of the visual stimuli, some studies have combined the auditory task with visual target stimuli. Finally, there have been attempts to record vMMN without any concurrent task and vMMN has been investigated using task-related stimuli. On one hand, it is important to note that even if the level of attentional control in vMMN studies is highly variable, the results of the various studies have been remarkably similar, since their overwhelming majority has reported negative-going deviant minus standard ERP components with posterior scalp distribution in the ~100–400 ms range. Nevertheless, it does not mean that strictly controlling attention is not required in future studies, since attentional effects might overlap with and confound components related to automatic mismatch processes. On the other hand, as the results of some studies show, vMMN is not independent of the characteristics of the ongoing task, but in this respect, the results are not unequivocal.

By varying the difficulty of a tracking task, Heslenfeld (2003) obtained identical vMMNs, but the amplitude of an anterior positivity decreased as a function of tracking difficulty. In an fMRI study, Yucel et al. (2007) reported reduced deviant-related posterior activity during a more difficult tracking task. Kimura et al. (2010b) investigated sequential regularity effects on vMMN and observed that vMMN-related activity to the rare stimuli of the regular patterns were absent in a conditions where participants attended to the regularity. Kimura and Takeda (2013) presented a set of bars in a passive oddball sequence, and varied the difficulty of a size discrimination task, where from time to time the fixation circle became smaller. Using an equal probability control the authors eliminated the earlier effect of deviant-related negativity. As a function of task difficulty the latency of the deviant-related negativity (vMMN) became longer (186, 195, and 226 ms, respectively). It seems that the difficulty of a task-set had a moderate effect on the speed of deviant processing. Task difficulty had no effect on vMMN amplitude. Kuldkepp et al. (2013) utilized motion direction stimuli and instructed participants to ignore or attend motion stimuli presented in the background. The authors found two distinguishable posterior vMMN components in the ignore condition, whereas in the attended condition a differential response was only observed in the later interval at frontal location. Kremláček et al. (2013) systematically varied attentional load (no-load, easy, and difficult) using a central number detection task also during presenting oddball sequences of visual motion direction stimuli. They found no effect of attentional load manipulation on vMMN amplitude.

In an MEG study by Kogai et al. (2011), vMMN responses elicited by undetected (masked) stimuli were recorded. The standard and deviant stimuli were gratings with various spatial frequencies. The authors obtained stronger responses to deviants in the 143–154 ms range even when deviant detection was below 6%. Perhaps it is safe to conclude that vMMN is a correlate of automatic processes, but these processes are not fully independent of the load and specificity of the ongoing task.

Another aspect of automaticity, namely processing capacity, was assessed by Czigler and Sulykos (2010). In this study reduced orientation-related vMMNs to peripherally presented bar-patterns were observed when the central task required orientation detection, and color-related vMMNs were also reduced if the central task required color detection. It seems plausible that sharing processing resources of structures involved in the primary attention task may have reduced the activity of the mechanisms underlying vMMN. In the field of visual attention research similar results were obtained within the framework of the dimensional weighting theory (Müller et al., 1995). The feature-specific effect implies a limit of the vMMN automaticity, and that *both overt attention and automatic change detection (predictive) processes might rely on the same or overlapping neural resources*. If the processing of task-relevant and irrelevant stimuli share certain common structures, and the former has a selective effect on the latter, then processes underlying vMMN are not fully autonomous. Importantly, in the study by Czigler and Sulykos (2010) the effect of shared capacity was due to the influence of a task-set (attend to orientation or attend to color), instead of the necessity of simultaneous stimulus processing. Thus, the influence on vMMN had to be originated by control processes.

The relationship between the stimuli regulating the ongoing behavior and the processing of irrelevant changes requires further investigations. This is because phenomena of visual attention, like contingent capture (e.g., Folk et al., 1992) may predict the facilitation of task-related dimensions, instead of the diminished activity within such dimensions. In summary, it is recommended to control for attentional effects as efficiently as possible, but taking into account also that highly demanding tasks may exhaust participants faster.

### The link between vMMN, veridical perception, and behavior

Automaticity is a key characteristic of the MMN response. Perceptual learning and the generation of perceptual prediction error responses have been demonstrated to occur in the absence of focused attention. Since behavior is usually linked to performance on the processing of task-relevant items, and vMMN stimuli are task-irrelevant, the issue of a relationship between vMMN and behavior is seldom investigated. However, just because information processing mechanisms operate independently of attention, it does not mean that they do not influence behavior. In fact, most of the information carried by the light entering the retina is processed “automatically” without conscious effort and relying on attentional resources (Velmans, 1991). The question arises whether vMMN mechanisms play a functional role in such automatic processes. As mentioned in Section Memory mismatch and refractoriness, the main function of System 1 in Kahnemann's framework is to maintain and update our predictive model of the world (Kahneman, 2011) and MMN is the neural correlate of the automatic detection of *unpredicted* changes in our visual environment carried out by System 1. That is, processes underlying the auditory and visual MMN seem to have key role in veridical perception. But how does veridical perception affect everyday behavior?

The auditory and visual MMN response is thought to reflect the important cognitive process of automatic stimulus discrimination (for reviews, see Kujala et al., 2007; Czigler and Pató, 2009; Näätänen et al., 2007, 2011; Kujala and Näätänen, 2010). A relationship between auditory MMN and behavioral measures of discrimination ability has been reported in several studies (Lang et al., 1990; Näätänen et al., 1993; Baldeweg et al., 1999; Desjardins et al., 1999; Amenedo and Escera, 2000; Kujala et al., 2001; Novitski et al., 2004; De Sanctis et al., 2009). It is generally accepted in the auditory MMN field that perceptual discrimination performance is strongly associated with MMN characteristics (amplitude and/or latency), e.g., increasing stimulus deviance increases MMN amplitude which correlates with higher discrimination rate. From a predictive point of view, perception involves inference about the causes of sensory input received by the brain. The fact that magnitude of prediction error response evoked by improbable events exhibits a relationship with behavioral measures of discrimination performance indicates that the efficiency of perceptual categorization may depend on the ability of the brain to infer upon the causes of sensory input. Automatic sensory discrimination reflected by auditory MMN is also associated with psychosocial functioning in healthy adults (Light et al., 2007) and has been suggested to serve as a gateway to higher order cognitive operations (Rissling et al., 2013). Similarly in the visual domain, vMMN has been argued to show automatic categorization processes based on fairly complex stimulus representation (Czigler, 2013).

It is uncommon in vMMN studies to collect behavioral data relevant to the processing of the vMMN-evoking stimuli. One reason is that usually a distractor task is employed in vMMN paradigms (as discussed in the previous section), where participants behaviorally respond, usually by pressing a button, to task-relevant stimuli. The distractor task serves the important purpose to eliminate potential effects of attention on ERPs to task-irrelevant standard and deviant stimuli. Applying a distractor task allows the experimenter to focus exclusively on effects of “surprise” or “deviance,” since brain responses to unattended and task-irrelevant stimuli are supposed to be uncontaminated by attentional and behavioral response-related activities. Thus, the standard and deviant ERPs in vMMN paradigms are usually task-irrelevant; consequently no behavioral data is collected during their recordings which could demonstrate the relevance of the processes underlying vMMN generation to behavioral functions. Another possible reason is that often low-level visual features are used to establish regularities in vMMN experiments (e.g., line orientation, spatial frequency) without obvious links to higher-level cognitive functions that are usually probed by behavioral tasks. Thus, the behavioral significance of vMMN responses, or the relationship between the vMMN response and behavioral measures is seldom demonstrated.

How can we obtain behavioral measures relevant to perceptual (cognitive) processes putatively related to vMMN processes, when vMMN is evoked by unattended and task-irrelevant events? The behavioral advantages brought about by automatic deviance detection systems (“primitive intelligence,” Näätänen et al., 2001) should be demonstrated in vMMN studies. To this end, one should show that there is a link between a vMMN property (e.g., amplitude, latency) and a behavioral index of performance in the cognitive domain where a regularity was used in a given experiment. To gain insight into how visual prediction error responses support veridical perception, we suggest that future studies should investigate the *relationship between visual mismatch responses and relevant behavioral measures*. Obtaining behavioral data (psychophysics, questionnaires, etc.) in separate protocols that assess functions putatively related to the vMMN-generating system is recommended.

Until now, only a few studies investigated the relationship between vMMN and behavior. In a study by Stefanics and Czigler (2012) laterality of hands was used to establish a regularity in the stimuli (e.g., pictures of right hands were repeated frequently (standard) with occasional pictures of left hands (deviant) interspersed in the stimulus stream). Preference of participants to use one hand over the other was measured by the Edinburgh handedness questionnaire. They found a significant relationship between handedness score and visual mismatch amplitude at the left fronto-temporal region for right-hand deviants, indicating that hand preference and MMRs to hands with unexpected laterality are related, however the exact nature of the relationship is not yet clear. In a recent study by Gayle et al. (2012) happy and sad faces were used to elicit vMMN in healthy individuals and autism spectrum personality traits were measured by the Adult Autism Spectrum Quotient (AQ). Smaller vMMN amplitudes to happy faces were associated with higher AQ score, and the authors suggested that vMMN evoked by unexpected emotional expressions may be a useful indicator of affective reactivity. Another recent study (Csukly et al., 2013) using emotional faces reported a correlation between vMMN amplitude to happy faces and emotion recognition performance as measured by the Ekman-test (Ekman and Friesen, 1976), both in healthy subjects and patients with schizophrenia.

The importance of auditory MMN-generating processes in supporting cognition and everyday behavior by veridical perception is highlighted in neurodevelopmental and psychiatric disorders where cognitive impairments are often accompanied by MMN deficits (for a review, see Näätänen et al., 2011). Numerous studies on developmental dyslexia used auditory MMN as an objective index of deficits in auditory information processing (Kujala and Näätänen, 2001). Furthermore, audiovisual training has been shown to enhance auditory cortical discrimination accuracy, as indexed by MMN, and concurrently improve reading skills in children with dyslexia (Kujala et al., 2001).

In schizophrenia research, one of the most replicable electrophysiological abnormalities is the reduced auditory MMN response (Umbricht and Krljes, 2005; Todd et al., 2012). MMN deficits are one of the features in schizophrenia that indicate severe abnormalities in fundamental brain processes of prediction and inference (Stephan et al., 2006). This is further corroborated by parallel evidence for a key role of NMDA receptors in auditory MMN generation and in the pathophysiology of schizophrenia (Umbricht and Krljes, 2005; Coyle, 2006; Javitt, 2009). Visual MMN studies with clinical samples are relatively rare (for a review, see Maekawa et al., 2012; Kremláček et al., in preparation) but they provide hints to a relationship between vMMN and various deficits. Urban et al. (2008) used deviant motion-direction and found attenuated vMMN in patients with schizophrenia, which was associated with medication dose, level of functioning and the presence of a deficit syndrome. A study by Maekawa et al. (2013) found attenuated vMMN to deviant windmill pattern stimuli with high spatial frequency in patients with bipolar disorders. Another recent vMMN study by Csukly et al. (2013) used deviant emotional expressions and found attenuated vMMN in schizophrenia patients which correlated strongly with decreased emotion recognition. These studies indicate a relationship between insufficient automatic processing of both lower-level (motion, spatial frequency) and higher-level (emotion) deviant characteristics and symptoms. A study by Wang et al. (2010) used vMMN to study orthographic processing skills in Chinese children with developmental dyslexia. They found reduced vMMN to moving gratings with deviant direction in the dyslexia group suggesting impaired visual discrimination processes, which might be related to reading deficits. Cléry et al. (2013b) used vMMN elicited by dynamic stimuli to study automatic sensory discrimination in children with autism spectrum disorder (ASD). They found an earlier visual MMR in children with ASD which the authors interpreted as a sign of hypersensitivity to visual deviancy. Although there are relatively few clinical vMMN studies yet, taken together, they suggest that impaired automatic visual discrimination might underlie or contribute to deficits in a variety of developmental and psychiatric syndromes.

The above examples illustrate that vMMN deficits are present in psychiatric and developmental disorders and that a correlative relationship between vMMN and specific behavioral indices has already been demonstrated in a handful of studies. The visual MMN seems to predict some aspects of behavior (such as personality traits, handedness, and emotion recognition skills) thus it might be a *potential biomarker in populations with deficits in specific cognitive domains*.

## Conclusions

Visual MMN similarly to auditory MMN is a promising basic and clinical research tool. Several studies confirmed that vMMN can be elicited by infrequent changes in lower- and higher-level attributes of simple and more complex stimuli. VMMN reflects automatic perceptual prediction error responses to events violating statistical regularities, and is a correlate of model update processes which likely operates through short term synaptic plasticity involving stimulus specific adaptation. In general, we recommend that future vMMN studies should take into account the issues regarding repetition suppression (refractoriness). We recommend using effective primary tasks to avoid attentional confounds. Finally, to show that vMMN obtained by violation of a regularity in a particular cognitive domain is not only an intriguing epiphenomenon we recommend investigating the relationship between vMMN attributes and discrimination performance in the cognitive domain relevant to the particular regularity.

### Conflict of interest statement

The authors declare that the research was conducted in the absence of any commercial or financial relationships that could be construed as a potential conflict of interest.

## Footnotes

^1^Note that Kahnemann is only using the distinction of System 1 and 2 as a metaphor of two agents to illuminate different aspects of human cognition.

^2^ For example, the free Matlab-based SHINE (Spectrum, Histogram, and Intensity Normalization and Equalization) toolbox offers functions to control low-level image properties (Willenbockel et al., 2010).

^3^ The cascadic control can also be viewed as a “roving standard” paradigm with predictable changes in pitch in two directions alternating in short, regular sequences. Strictly speaking, in the oddball sequence pitch change has a low probability, whereas in the cascadic control a certain change in a given direction has a high probability. One might argue that difference between the response to the oddball deviant and its cascadic control might not only reflect differences in prediction errors but also activity related to fulfilled predictions.
